# LDHB Deficiency Promotes Mitochondrial Dysfunction Mediated Oxidative Stress and Neurodegeneration in Adult Mouse Brain

**DOI:** 10.3390/antiox11020261

**Published:** 2022-01-28

**Authors:** Jun Sung Park, Kamran Saeed, Myeung Hoon Jo, Min Woo Kim, Hyeon Jin Lee, Chan-Bae Park, Gwang Lee, Myeong Ok Kim

**Affiliations:** 1 Division of Life Science and Applied Life Science (BK21 FOUR), College of Natural Sciences, Gyeongsang National University, Jinju 52828, Korea; jsp@gnu.ac.kr (J.S.P.); kamran.biochem@gnu.ac.kr (K.S.); audgns1217@gnu.ac.kr (M.H.J.); mwkim0322@gnu.ac.kr (M.W.K.); lhj4912@gnu.ac.kr (H.J.L.); 2 Department of Otolaryngology, Ajou University School of Medicine, Suwon 16499, Korea; pcbkaist@gmail.com or; 3 Department of Physiology, Ajou University School of Medicine, Suwon 16499, Korea; glee@ajou.ac.kr; 4 Alz-Dementia Korea Co., Jinju 52828, Korea

**Keywords:** Lactate dehydrogenase-B, mitochondrial dysfunction, oxidative stress, inflammation, p-AMPK/Sirt1/PGC-1alpha signaling, osmotin, neurodegeneration

## Abstract

Age-related decline in mitochondrial function and oxidative stress plays a critical role in neurodegeneration. Lactate dehydrogenase-B (LDHB) is a glycolytic enzyme that catalyzes the conversion of lactate, an important brain energy substrate, into pyruvate. It has been reported that the LDHB pattern changes in the brain during ageing. Yet very little is known about the effect of LDHB deficiency on brain pathology. Here, we have used Ldhb knockout (*Ldhb*^−/−)^ mice to test the hypothesis that LDHB deficiency plays an important role in oxidative stress-mediated neuroinflammation and neurodegeneration. LDHB knockout (*Ldhb*^−/−^) mice were generated by the ablation of the *Ldhb* gene using the Cre/loxP-recombination system in the C57BL/6 genetic background. The *Ldhb*^−/−^ mice were treated with either osmotin (15 μg/g of the body; intraperitoneally) or vehicle twice a week for 5-weeks. After behavior assessments, the mice were sacrificed, and the cortical and hippocampal brain regions were analyzed through biochemical and morphological analysis. *Ldhb*^−/−^ mice displayed enhanced reactive oxygen species (ROS) and lipid peroxidation (LPO) production, and they revealed depleted stores of cellular ATP, GSH:GSSG enzyme ratio, and downregulated expression of Nrf2 and HO-1 proteins, when compared to WT littermates. Importantly, the *Ldhb*^−/−^ mice showed upregulated expression of apoptosis mediators (Bax, Cytochrome C, and caspase-3), and revealed impaired p-AMPK/SIRT1/PGC-1alpha signaling. Moreover, LDHB deficiency-induced gliosis increased the production of inflammatory mediators (TNF-α, Nf-ĸB, and NOS2), and revealed cognitive deficits. Treatment with osmotin, an adipoR1 natural agonist, significantly increased cellular ATP production by increasing mitochondrial function and attenuated oxidative stress, neuroinflammation, and neuronal apoptosis, probably, by upregulating p-AMPK/SIRT1/PGC-1alpha signaling in *Ldhb*^−/−^ mice. In brief, LDHB deficiency may lead to brain oxidative stress-mediated progression of neurodegeneration via regulating p-AMPK/SIRT1/PGC-1alpha signaling, while osmotin could improve mitochondrial functions, abrogate oxidative stress and alleviate neuroinflammation and neurodegeneration in adult *Ldhb*^−/−^ mice.

## 1. Introduction

Mitochondria play an important role in cellular metabolism; generating energy and its proficient function is paramount in the neuronal system. However, with aging, the bioenergetics’ state, characterized by impaired mitochondrial functions, is associated with not only the diminished ATP formation but also promotion of various neurodegenerative diseases, including Alzheimer’s disease and Parkinson disease, through retrograde signaling, such as reactive oxygen species (ROS) and calcium dyshomeostasis. The compromised brain mitochondrial functions impair cellular redox state through abnormal intermediation of the intracellular antioxidant defense system [1,2]. Thus, the impaired mitochondria with advanced age render neurons more susceptible to stresses of various endogenous and exogenous origins [3].

Lactate dehydrogenase (LDH) is an enzyme involved in the interconversion of lactate and pyruvate. LDH is a tetrameric-protein composed of two subunits, LDHA and LDHB, which assemble into five different isozymes (LDH1-LDH5) [4] expressed in tissues with high-energy demands such as skeletal muscle, heart muscle [5], and nervous system [6]. The brain requires a continuous source of energy as it has minimal glucose storage capabilities [7]. Neurons are dependent on the lactate derived from astrocytes as energy substrate via monocarboxylate transporters (MCTs); the notion is explained by the astrocyte-neuron lactate shuttle (ANLS) theory [8,9,10,11]. Downregulation of LDHB expression disrupts the conversion of lactate into pyruvate, impairing mitochondrial ATP production and suppressing oxidative phosphorylation [12,13]. During aging, the expression pattern of *LDH* genes (*LDHA* and *LDHB*) changes, instigating mitochondrial impairment [14].

Adiponectin (APN) is an insulin-sensitizing adipokine that regulates energy metabolism. APN improves mitochondrial functions and increases oxidative capacity in the skeletal muscles [15]. The biological actions of APN are predominantly mediated through APN receptors (AdpoR1 and AdipoR2) [16,17]. The AdipoR1 expression is ubiquitous in the rat brain [18]. AdipoR1 positively regulates and protects neuronal cells and neural stem cells after nervous system injury [19]. The regulatory actions of APN are attributed to the activation of 5’ AMP-activated protein kinase (AMPK) mediated downstream, signaling through APN receptors [17,20]. AMPK function as an energy sensor and maintain cellular energy hemostasis and metabolic pathways [21]. In the brain, the energetic stress and mitochondrial insult instigate AMPK phosphorylation, which mediates a cellular response and synchronizes multiple features of mitochondrial biology [22] through downstream signaling, including the activation of peroxisome proliferator-activated receptor-γ coactivator-1a (PGC-1α) [23].

PGC-1α plays an important role in cellular energy metabolism. Recent studies accentuated the role of PGC-1α as a central regulator of mitochondrial biogenesis and cellular respiration [24]. Notably, the role of PGC-1α in neurodegeneration has been well-studied [25]. The reduced expression pattern of PGC-1α correlates with brain mitochondrial dysfunction and oxidative stress in patients with the neurodegenerative disease [26,27]. Likewise, PGC-1α null mice display an increased sensitivity to the neurodegenerative response of oxidative stress, and its expression protects neuronal cells by suppressing ROS production through induction of ROS-detoxifying enzymes [28]. Importantly, PGC1-α regulates neuronal mitochondrial density [29] and interacts with nuclear respiratory factors to enhance mitochondrial functions [30].

Osmotin, a stress-responsive natural plant protein, mimics mammalian adiponectin [31]. Osmotin initiates downstream signaling by binding to PHO36 receptor, which is a mammalian homolog of human AdipoR1 [32]. Previously, we confirmed that osmotin activates AdipoR1/AMPK axis to alleviate neuropathological deficits in amyloid precursor protein/presenilin 1 (APP/PS1) mice [33], relieve oxidative stress, improve the mitochondrial system, and protect the neonatal brain from neuronal apoptosis, in the developing rat brain, against ethanol-induced neurodegeneration [34]. Herein, we have unraveled the role of LDHB-driven alterations in the brain. We have speculated that the deficiency of the LDHB enzyme leads to mitochondrial dysfunction associated with increased brain oxidative stress, concomitant with neuroinflammation and neurodegeneration. At the same time, the administration of osmotin alleviates these discrepancies, probably, by upregulating p-AMPK/SIRT1/PGC-1α signaling, improved cellular-redox hemostasis, and cellular ATP levels in the cortex and the hippocampal brain region. To address this challenge, we have generated and used *Ldhb*^−/−^ mice to verify the role of LDHB in promoting brain oxidative stress-mediated neuroinflammation and neurodegeneration.

## 2. Materials and Methods

### 2.1. Generation of Ldhb Null Mice

LDHB null mice were generated as we have previously reported [35]. The mice were generated by the ablation of the Ldhb gene using the Cre/loxP-recombination system in the C57BL/6 genetic background. Production of homozygote (Ldhb^−/−^) mice was done at Ajou University School of Medicine, South Korea. The resulting mice were transferred to Gyeongsang National University. All mice were adapted for 1 week under 12-h light/dark conditions at 23–25 °C, with 60 ± 10% humidity, and were provided with free access to water and food in the university animal laboratory. All procedures were approved and conducted under the guidelines of the animal ethics committee of the Division of Applied Life Sciences, Gyeongsang National University, South Korea (Approval number: GNU-200331-M0020).

### 2.2. Experimental Subjects and Drug Administration

For experiments, the same aged-matched animals were randomly divided into three different groups (*n* = 4/group), Saline treated wild type, Ldhb^−/−^ + Saline treated, and Ldhb^−/−^ + Osmotin treated groups. Osmotin was administered at dose concentration 15 μg/g of body weight in saline solution, twice a week for 5-weeks, via intraperitoneal injection. Animals had free access to food and fresh water. Mice were anesthetized and decapitated at the age of 24 months. All experiments performed followed the guidelines and principles of the Animal Ethics Committee (IACUC) (Approval ID: 125) from the Division of Applied Life Sciences at Gyeongsang National University (GNU), South Korea.

### 2.3. Behavioral Analysis

The spatial learning and memory functions were examined using a Y-Maze and Morris water maze (MWM) test. All animal trials were recorded and route-tracked with automated-tracking software (SMART, Panlab Harward-Apparatus, Bioscience Company, Holliston, MA, USA), and statistical analysis of the data was performed using GraphPad Prism (ver.8.0.2, San Diego, CA, USA) software.

The Y-maze apparatus consists of three identical arms of equal length (50 cm L × 20 cm H × 10 cm W) at a 120° angle within each arm. To check spontaneous alterations or the innate curiosity of animals to explore the novel areas, the mice were placed in the center of Y-maze at the arms junction site and freely allowed to explore the apparatus for 8 min. An entry of an animal was considered when all four limbs were inside the arm. The total number of arm entries and successive triplets were recorded. The [%] spontaneous alterations were measured as 100× [consecutive entries into three different arms)/the total number of arm entries − 2]. The % spontaneous alternations correlated positively with spatial working and memory function.

The MWM consists of a circular metal pool (1 m dia. × 0.4 m H) with a uniform interior, filled ~65% with water (25 ± 1 °C) opacified by supplementing with non-toxic tempera paint. A circular platform (0.1 m dia. × 0.14 m H) was submerged ~1 cm below the water’s surface in one of the four arbitrary pool quadrants for rescue purposes. The automated camera was fixed just above the center of the maze on the ceiling to monitor and document the animal routes during a behavioral test. A day before training, mice received an acclimatization period and were allowed to swim in the water pool and rested on the platform for 30 s. Each mouse (*n* = 4/group) was then subjected to a consecutive 4-day training period with 4 trials (60 s/trial) per day. During the training period, the mice were placed in one of the quadrants and were allowed to search the submerged platform. The animals that failed to locate the platform in the permitted time were physically guided and placed on the platform for 10 sec before being removed. For each trial, the starting location was changed from one arbitrary quadrant to another, and the escape latencies were measured for all days. On the 5th day after training, a probe test was conducted without a platform in the pool to check the memory retention. Animals were allowed to swim from one of the quadrants for 60 sec, and both the number of times the mice crossed and the time spent in the formal platform zone were measured.

### 2.4. Brain Tissue Collection and Sample Preparation

After cognition assessments, the mice were anesthetized with Rompun (M&S Korea) mixed with Zoletil (M&S Korea) (1:2) (administered; 0.5 mL per 100 g of body weight). For biochemical analysis, the brains were immediately removed, and the hippocampi and cortices regions were dissected out carefully and flash-frozen with liquid nitrogen. For protein extraction, the tissues were mechanically homogenized in PRO-PEP extraction solution on the surface of the ice and centrifuged at 13,000 rpm for 30 min at 4 °C. The supernatants were collected and analyzed by SDS-PAGE. For immunostaining and morphological analysis, the animals were perfused transcardially with normal saline and then, subsequently, with 4% paraformaldehyde (PFA). The brains were removed cautiously, submerged, and fixed at 4 °C in ice-cold PFA for 72 h and were then rinsed with filter-Phosphate-buffered saline (f-PBS) and placed in 20% sucrose solution for 48 h before freezing in OCT compound (Tissue-Tek O.C.T compound medium, Sakura Finetek USA, Inc., Torrance, CA, USA #4583). Serial 14 μm coronal tissue sections were cut on CM 3050C cryostat (Leica, Nussloch, Germany) microtome and thaw-mounted on gelatin-coated slides.

### 2.5. Immunoblotting, Immunofluorescence and Immunohistochemistry

Western Blot was performed as we have previously reported [33]. The protein quantitation was performed for immunoblotting using Bio-Rad protein assay (Bio-Rad Laboratories, Hercules, CA, USA). Equal quantities (20 μg) of protein were fractioned by SDS-PAGE, followed by transfer to polyvinylidene difluoride (PVDF) membrane. To reduce non-specific bindings, the membranes were blocked in skim milk (5% in TBST) for 1h and incubated overnight (at 4 °C) with primary antibodies to reveal the protein of interest. β-actin levels were used as an internal control.

For immunofluorescence, the fixed brain tissue/sections were washed twice (eight min/wash) with filter-phosphate buffered saline (f-PBS). The slides were then treated with proteinase K for 5 min, and washed again twice for 5 min, each with f-PBS. The slides were then blocked with 5% goat serum (diluted with 0.3% Triton X-100 in f-PBS) for 1 h at room temperature before being rinsed with f-PBS. The slides were incubated overnight (at 4 °C) with primary antibody and then, with a secondary TRITC- or FITC-conjugated antibody for 90 min at room temperature. After incubation, the secondary antibody solution was carefully removed, followed by washing for 10 min with f-PBS and counter-staining with DAPI. The mounting medium (Dako North America, Inc. 6392 Via Real Carpinteria, CA, 93013 USA) was applied on the slides and cover-slipped. Images were acquired with laser-scanning confocal microscopy (FluoView FV 1000; Olympus, Tokyo, Japan). The data sets from both techniques were analyzed and plotted using ImageJ software (v. 1.50, NIH, Bethesda, MD, USA) and GraphPad Prism (ver. 8.0).

Immunohistochemistry for LDHB was performed by initially washing the brain slides with f-PBS (5 min), followed by incubation with proteinase K (20 mg/mL) for 10 min. The slides were then treated with 9:1 of methanol and H_2_O_2_ solution for 10 min, followed by blocking with normal goat serum in 5% BSA, containing Triton x-100(0.03%) at room temperature for 1.5 h. The brain slices were then incubated overnight at 4 °C with primary antibody (anti-LDHB) followed by washing and incubation with secondary biotinylated anti-rabbit IgG for 2 h. The sections were then treated with ABC solution for 1 h at room temperature. After DAB-chromogen application, the slides were washed with f-PBS (5 min, 2 times) and dehydrated in 50, 70, and 95% ethanol solution, respectively, for 1 min each. The tissues sections were then cleared in xylene solution for 5 min and mounted. The brain morphology of stained slides was analyzed using a microscope (Zeiss Axioskop 2 Plus, Oberkochen, Germany). The primary and secondary antibodies used are shown in Table 1.

### 2.6. Nissl Staining

Nissl staining was performed to visualize the Nissl bodies to determine the morphological and pathological changes in the brain neuronal structure [36]. In brief, the slides containing brain tissue were washed with water, to remove the salt’s residue, and stained for 8–10 min with the warm cresyl violet solution (0.5%), supplemented with a few drops of glacial acetic acid. The slides were quickly rinsed with MilliQ water to remove excess stain and then progressively dehydrated in 70, 95, and 100% ethyl alcohol for 15 min. The brain slices were then cleared twice in xylene, for 5 min each, before being mounted with a non-fluorescence permanent mounting medium and a coverslip. For morphological visualization, TIF images were acquired with a fluorescence optical light microscope, and the number of surviving neuronal cells in the hippocampi and cortices area was analyzed with ImageJ software.

### 2.7. ROS, Lipid Peroxidation (MDA), ATP and GSH Assays

The ROS assay was performed, as previously described, with minor modification [37]. This assay is based on the formation of 2′7′ dichlorofluorescein (DCF) from the oxidation of 2′7′-dichlorodihydrofluorescein diacetate (DCFH-DA) (Santa Cruz, Dallas, USA, CAS #4091-99-0). The homogenates of the cortex and hippocampus were diluted with a concentration of 1:20 in ice-cold Lock’s buffer to yield 2.5 mg/tissue/500 μL. Next, the 1 mL of the Lock’s buffer mixture (pH ± 7.4), containing 0.2 mL of homogenates, was incubated with 10 mL of DCFH-DA (5 mM) solution, at room temperature for 30 min, to make fluorescent product dichlorofluorescein (DCF). For the calculation of DCF formation, parallel blanks were measured without homogenate. The ROS levels were measured using a microplate reader, and the data are expressed as DCF formed (pmol)/the amount of protein (mg).

Quantification of lipid peroxidation was assessed by measuring malondialdehyde (MDA) concentration using an MDA colorimetric/fluorometric assay kit (Bio Vision, San Francesco, CA, USA, Cat# K739–100) according to manufacturer protocols.

The glutathione (GSH) and GSH: oxidized GSH (GSSG) levels of the brain homogenates were measured using a fluorometric GSH assay kit (Bio-Vision Inc., 155 S. Milpitas Boulevard, Milpitas, CA 95035, USA), according to the company’s approved guidelines.

For the detections of ATP levels, ATP Assay Kit (Colorimetric/Fluorometric) (ab83355) was used, according to the company’s protocol.

Fluorescence intensities of the assays were recorded (ROS: Ex/Em = 484/530 nm, MDA: Ex/Em = 532/553 nm, ATP: Ex/Em = 535/587 nm and GSH: Ex/Em = 340/420 nm and) using 96-well fluorescence microplate reader ApoTox-Glo™ (Triplex-Assay, Promega, Madison, WI, USA).

### 2.8. Statistical Analysis

Values are illustrated as means ± standard errors of the mean (SEM). All the data sets were plotted and statistically analyzed using one-way ANOVA, followed by Tukey’s post-hoc test, using computer-based GraphPad Prism software (ver.8.0, San Diego, CA, USA). The *p* values that presented less than 0.05 were considered statistically significant between the two groups. Statistical analysis is conferred in Figure legends.

## 3. Results

### 3.1. Generation of Ldhb KO mice

**Figure 1:**
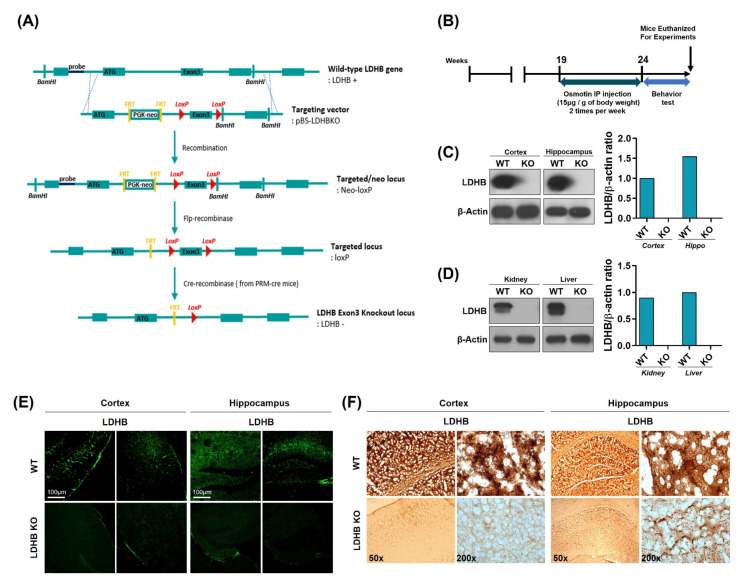
Schematic diagram showing the generation of *Ldhb*^−/−^ mouse, experimental design, and verification of *Ldhb*^−/−^ model. (**A**) Schematic diagram illustrating the Cre-loxP and Flp-FRT system using pBS-*Ldhb* KO vectors. *Ldhb*^−/−^ mice were generated through gene knockout at the exon 3 loci. (**B**) Schematic diagram of experimental design, showing the period of osmotin treatment in *Ldhb*^−/−^ mice and behavior test. (**C**,**D**) The Western blot analysis showing the expression of LDHB in the cortex, hippocampus, kidney, and liver of *Ldhb*^−/−^ and WT mice. β-actin was used as a loading control. The bands were quantified using ImageJ software, and the differences are represented by histograms. (**E**) Confocal microscopic images showing the expression of LDHB in the cortex and hippocampus of *Ldhb*^−/−^, WT mice. (**F**) Immunohistochemistry staining for LDHB in the cortex and hippocampus of *Ldhb*^−/−^, WT mice.

Complete allelic *Ldhb*^−/−^ mouse strains were generated as we have previously reported [35]. Briefly, *Ldhb* floxed (*Ldhb* loxP/^+^) mice were generated through recombination using pBS-Ldhb (Liu, Genome Res) KO targeting vector. These mice were then crossed with cre-transgenic mice in the C57/Bl6 background, expressing cre recombinase under the control of protamine1 promoter (PRM-cre) to generate *Ldhb*^+/−^ offspring. The *Ldhb*^−/−^ mice were then generated by breeding heterozygous (*Ldhb*^+/−^) pairs. The schematic (Figure 1A) describes the generation of the *Lhdb* gene knockout locus. *Ldhb* KO mice were treated with osmotin intraperitoneal injections two times a week, for 5 weeks, from 19 to 24 weeks of age. The osmotin was administered at a dose of 15 μg per 1g of body weight. After behavioral tests, mice were euthanized for experiments (Figure 1B). The loss of LDHB expression was confirmed in both the brain and periphery. As expected, the immunoblot results revealed no expression pattern of LDHB enzyme in the cortex and hippocampal brain homogenates of the *Ldhb* KO mice (Figure 1C). Likewise, no detectable LDHB expressions were found in the kidney and liver homogenates of LDHB deficient mice (Figure 1D). Immunofluorescence and immunohistochemistry further verified the loss of LDHB protein expression in the cortical and hippocampal brain slices of the *Ldhb*^−/−^ mice (Figure 1E,F). These findings confirm the generation of complete homozygous LDHB null mice.

### 3.2. Ldhb KO Mice Display Increased Brain Oxidative Stress and Redox Dyshomeostasis

**Figure 2:**
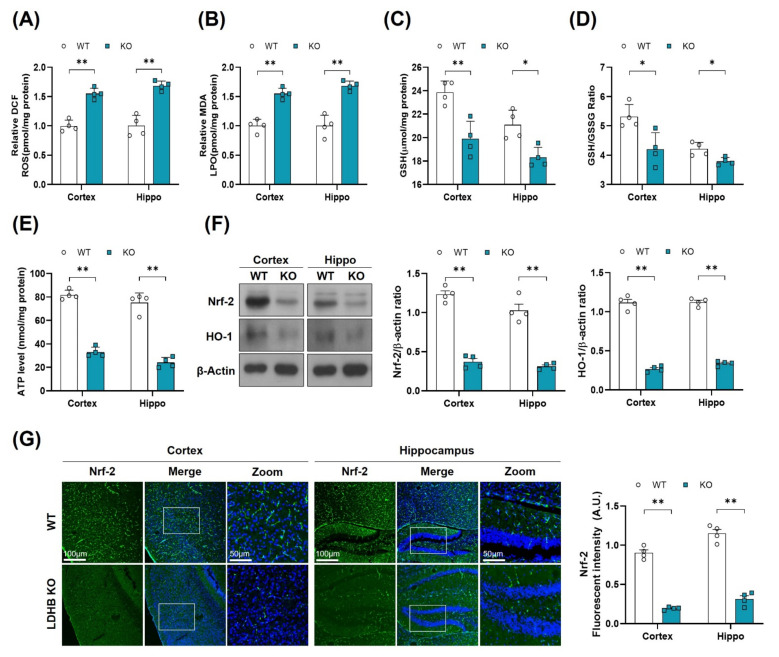
LDHB deficiency-induced oxidative stress in adult mouse brain. (**A**,**B**) Analysis of the generation of ROS and production of LPO in the cortex and hippocampus regions of *Ldhb*^−/−^ and WT mice. (**C**,**D**) The graphs represent the levels of glutathione (GSH) and GSH/GSSG ratio in the hippocampi of adult *Ldhb*^−/−^ and WT mice. (**E**) Quantitative analysis of cellular ATP levels in the cortical and hippocampal brain tissue lysate in the experimental groups. (**F**) Western blot analysis of Nrf2 and HO-1 in the cortex and hippocampus of adult mouse brains. The bands were quantified using ImageJ software, and the differences are represented by histograms. β-actin was used as a loading control. (**G**) Confocal immunofluorescence photomicrographs of Nrf-2 reactivity in the cortex and hippocampus of the experimental groups. The density values are relative to the WT group and expressed in arbitrary units (A.U.). The data are presented as the mean ± SEM of 4 mice per group, * *p* ≤ 0.05, ** *p* ≤ 0.01.

There is a wealth of literature demonstrating the physiological and pathological evidence that compromised mitochondrial function and dynamic plays important roles in aging and the pathogenesis of neurodegenerative diseases [38]. To determine whether *Ldhb*^−/−^ mice exhibit mitochondrial dysfunctions, we measured the ATP levels, oxidative stress parameters, and endogenous antioxidant markers, in both the cortex and hippocampus, compared to control littermates. The *Ldhb*^−/−^ mice displayed enhanced reactive oxygen species (ROS) and increased lipid peroxidation (LPO), as revealed by higher DCF fluorescence and MDA content, respectively, in the cortex and the hippocampal brain homogenates (Figure 2A,B). Moreover, the brain homogenates of *Ldhb* KO mice presented depleted values of GSH and GSH: GSSG ratio (Figure 2C,D), as well as low level of extracellular ATP (Figure 2E) when compared to the wild type (WT) group. Moreover, immunoblot quantification revealed a significant downregulation of Nrf-2 and HO-1 expressions in the cortex and hippocampal brain regions compared to the control mouse cohort (Figure 2F). The immunofluorescence analysis of brain slices of *Ldhb* KO mice further invigorated the loss of Nrf-2 immunoreactivity in the cortex and within the hippocampal-DG region (Figure 2G). Together, these results demonstrate that LDHB deficiency causes mitochondrial dysfunctions and promotes brain oxidative stress and cellular-redox dyshomeostasis in adult mice.

### 3.3. Ldhb KO Mice Exhibit Neuronal Loss and Neurodegeneration

**Figure 3:**
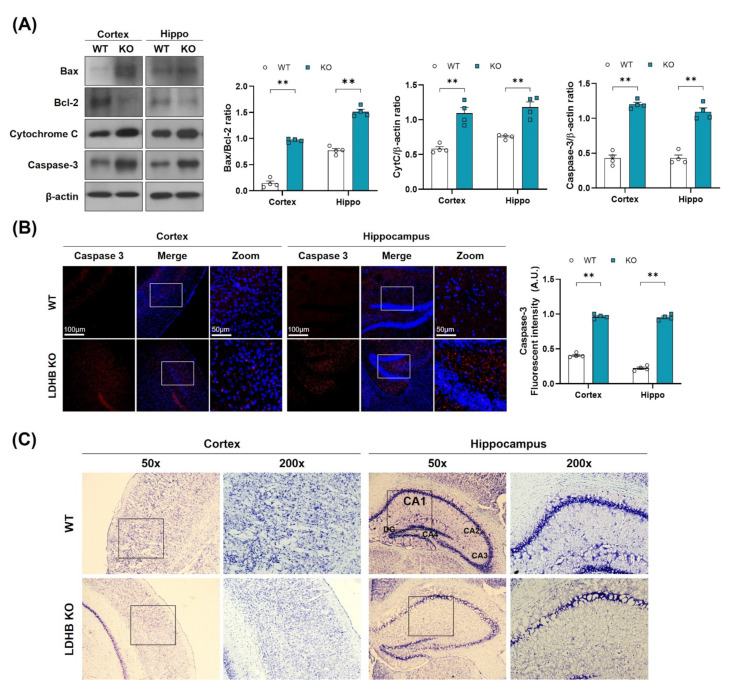
LDHB deficiency-induced apoptotic cell death in the cortex and hippocampus of the experimental groups. (**A**) Western blot analysis of Bax, Bcl-2, Cytochrome C and Caspase-3 in the cortex and hippocampus of *Ldhb*^−/−^ and WT mice. The bands were quantified using ImageJ software, and the differences are represented by histograms. β-actin was used as a loading control. (**B**) Confocal immunofluorescence photomicrographs of Caspase-3 reactivity in the cortex and hippocampus of the experimental groups. The density values are relative to the WT group and expressed in arbitrary units (A.U.). The data are presented as the mean ± SEM of 4 mice per group, ** *p* ≤ 0.01. (**C**) Photomicrograph of LDHB Nissl staining in the cortex and hippocampus region of the mouse brain.

As oxidative stress and mitochondrial dysfunction are likely to promote neuronal loss, as observed in many neurodegenerative diseases [39], we, therefore, hypothesized that enhanced ROS might be associated with cell death and neurodegeneration in *Ldhb* KO mice. To investigate this notion, we analyzed the neuro-apoptotic protein expressions in the cortex and hippocampus brain homogenates. We observed a significant upregulation in Bax/Bcl-2 ratio, cytochrome C, and caspase-3 protein expressions, leading to cell death and apoptosis in *Ldhb* KO mice (Figure 3A). Likewise, the immunofluorescence analysis also illustrated a prominent increased caspase 3 immunoreactivity in the cortex and hippocampal brain region of *Ldhb* KO mice compared to control littermates (Figure 3B). Furthermore, the cresyl violet stained brain slices of LDHB deficient mice also displayed neuronal degeneration in the cortex and within the hippocampal CA-1 region, as indicated by fewer, and sparsely arranged, Nissl substances compared to WT, which presented deeper and dark stained Nissl bodies with higher density (Figure 3C). These data indicate that loss of LDHB is associated with neuronal degeneration and cell death.

### 3.4. Osmotin Improved Mitochondrial Functions via Regulating the p-AMPK/SIRT1/PGC-1α Axis in Ldhb KO Mice

**Figure 4:**
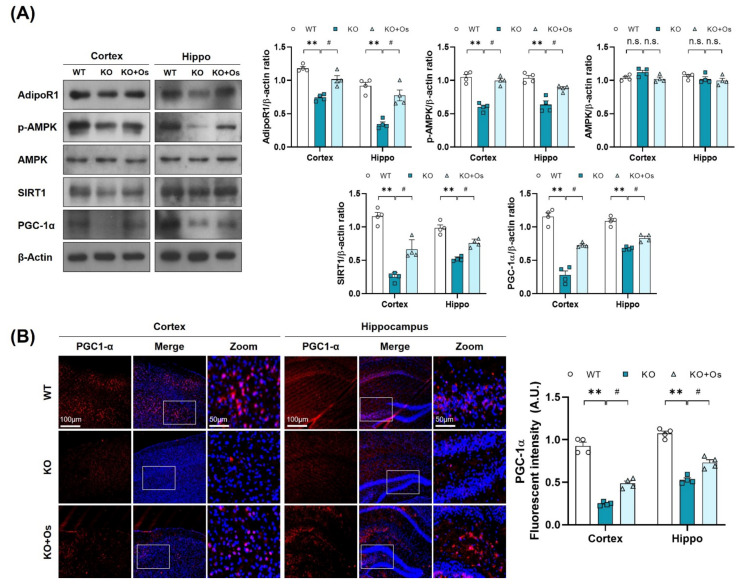
Treatment with osmotin upregulated p-AMPK/SIRT1/PGC-1alpha signaling, reduced by LDHB deficiency. (**A**) Western blot analysis of AdipoR1, p-AMPK, AMPK, SIRT1, and PGC-1alpha in the cortex and hippocampus of the experimental groups. The bands were quantified using ImageJ software, and the differences are represented by histograms. β-actin was used as a loading control. (**B**) Confocal immunofluorescence photomicrographs of PGC-1alpha immunoreactivity in the cortex and hippocampus of the experimental groups. The data is presented as the mean ± SEM of 4 mice per group and expressed in arbitrary units (A.U.). ** *p* ≤ 0.01, ^#^ *p* ≤ 0.05, n.s = not significant.

Since p-AMPK is an energy sensor molecule, it coordinates positively with Sirt1 to regulate PGC-1α and improve mitochondrial functions [21]. We thereby determined whether osmotin treatment would trigger the p-AMPK/SIRT1 axis to activate PGC-1α in *Ldhb* KO mice. Immunoblot results of *Ldhb*^−/−^ mice revealed reduced expressions of AdipoR1 and were associated with downregulated p-AMPK, SIRT1, and PGC-1α protein expression in cortical and hippocampal brain homogenates compared to the age-matched WT group (Figure 4A). Immunofluorescence analysis of the *Ldhb* KO brain slices further invigorated the downregulation of PGC-1α immunoreactivity within the cortex and hippocampal-DG region (Figure 4B). However, osmotin treatment enhanced AdipoR1 expression and was accompanied by rapid phosphorylation of AMPK (Thr172), and it increased SIRT1 expression, indicating a probable activation of AdipoR1/p-AMPK/SIRT1 signaling (Figure 4A). The treatment with osmotin also upregulated the PGC-1α expression and immunoreactivity, as revealed by both immunoblot and immunofluorescence analysis, respectively, in the cortex and hippocampal brain region (Figure 4A,B). These data suggest that a *Ldhb* KO mouse displays suppression of PGC-1α protein expression, probably associated with diminished p-AMPK/SIRT1 signaling, while osmotin treatment may instigate the p-AMPK/SIRT1 signaling to regulate PGC-1α to alleviate mitochondrial dyshomeostasis observed in *Ldhb* KO mice.

### 3.5. Osmotin Alleviates Brain Oxidative Stress and Neurodegeneration in Ldhb KO Mice

**Figure 5:**
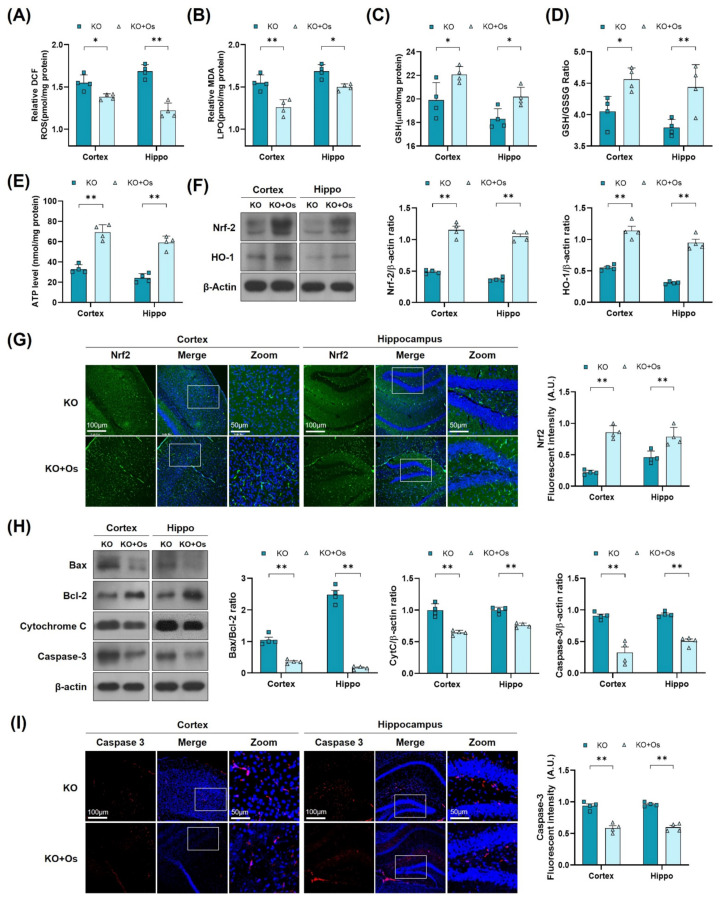
Osmotin alleviates oxidative stress and apoptosis, induced by LDHB deficiency in the cortex and hippocampus. (**A**,**B**) Analysis of the ROS generation and LPO production in the cortex and hippocampus of *Ldhb*^−/−^ and osmotin treated *Ldhb*^−/−^ mice. (**C**–**E**) The representative histograms show the glutathione (GSH) levels, GSH/glutathione disulfide (GSH/GSSG) ratio, and ATP levels in the brain tissue lysate of the adult mice. (**F**) Western blot analysis of Nrf2 and HO-1 in the cortex and hippocampus of adult mouse brains. (**G**) Confocal immunofluorescence photomicrographs of Nrf-2 reactivity in the cortex and hippocampus of *Ldhb*^−/−^ and Osmotin-treated groups. (**H**) Western blot analysis of Bax, Bcl-2, Caspase-3, and Cytochrome C in the cortex and hippocampus of adult mouse brains. (**I**) Confocal immunofluorescence photomicrographs of Caspase-3 reactivity in the cortex and hippocampus of *Ldhb*^−/−^ and Osmotin-treated groups. Western blot bands were quantified using ImageJ software, and the differences are presented by histograms. β-actin was used as a loading control. The density values are relative to the KO group and expressed in arbitrary units (A.U.). The data are presented as the mean ± SEM of 4 mice per group, * *p* ≤ 0.05, ** *p* ≤ 0.01.

To further determine if osmotin treatment would reduce the brain oxidative stress, improve mitochondrial functions, and protect against the neurodegeneration in LDHB deficient mice, we performed LPO, ROS GSH, and ATP assay, along with immunoblots and immunofluorescence analysis, of the cortical and the hippocampal brain regions. Both ROS and LPO assays demonstrated a decrease in oxidative stress, as revealed by a loss in DCF fluorescence and MDA contents, respectively (Figure 5A,B), whilst the GSH and GSH: GSSG levels were increased in osmotin treated *Ldhb* KO brain compared to saline-treated *Ldhb* KO mice (Figure 5C,D). Moreover, osmotin enhanced extracellular ATP levels in *Ldhb*^−/−^ mice (Figure 5E). Besides, osmotin treatment of *Ldhb* KO mice significantly enhanced Nrf2 expression and immunoreactivity, as well as HO-1 protein expression in comparison to saline-treated *Ldhb*^−/−^ mice (Figure 5F,G). Likewise, treatment with osmotin also improved the mitochondrial system by downregulating the expression of Bax/Bcl-2 ratio, alleviating cytochrome C release and abolishing cell death, as visualized by deescalated caspase3 expression and immunoreactivity, when compared to the saline-treated LDHB deficient mice (Figure 5H,I). Together, these results demonstrate that osmotin treatment improves mitochondrial bioenergetics and cellular-redox homeostasis by increasing the expression of endogenous cytoprotective-enzymes (HO-1 and GSH) and ATP levels to reduce ROS and rescue cell death and neurodegeneration.

### 3.6. Osmotin Impedes Gliosis and Neuroinflammatory Mediators in Ldhb KO Mice

**Figure 6:**
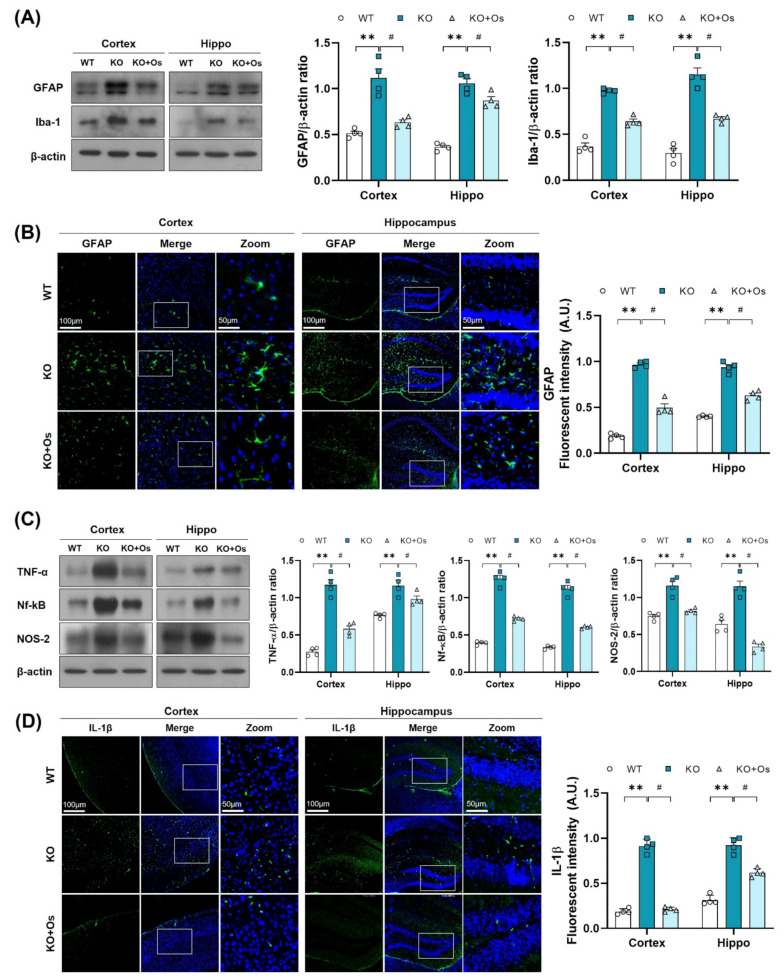
Osmotin alleviates immune response induced by LDHB deficiency in the cortex and hippocampus. (**A**,**C**) Western blot analysis of GFAP, Iba-1, TNF-α, Nf-ĸB, and NOS-2 in the cortex and hippocampus of adult mouse brains. The bands were quantified using ImageJ software, and the differences are represented by histograms. β-actin was used as a loading control. (**B**,**D**) Confocal immunofluorescence photomicrographs of GFAP and IL-1β reactivity, respectively, in the cortex and hippocampus of the experimental groups. The density values are relative to the WT group and expressed in arbitrary units (A.U.). The data are presented as the mean ± SEM of 4 mice per group, ** *p* ≤ 0.01, ^#^ *p* ≤ 0.05.

Mitochondrial oxidative damage is strongly associated with neuroinflammation [40] and glial cells activation, which may contribute to the inflammatory response and play an important role in the development of neurodegenerative diseases [41]. Therefore, we further investigated whether the *Ldhb* deficient mice display glial cell reactivity. Immunoblot results revealed enhanced gliosis, as observed by the upregulated expression of GFAP and IBA in the cortical and hippocampal brain homogenates of LDHB deficient mice, compared to WT littermates (Figure 6A). Immunofluorescence analysis further corroborated the presence of reactive astrocytes, as indicated by the increase in GFAP immunoreactivity in the *Ldhb* KO brain slices (Figure 6B). Remarkably, osmotin treatment significantly alleviated gliosis, as illustrated by the downregulated IBA and GFAP protein expression and a prominently reduced GFAP immunoreactivity in the cortex and hippocampal brain regions, compared to the WT group (Figure 6A,B). Since LDHB deficient mice displayed a modulation of glial activity, we further investigated the expression of various brain inflammatory mediators. Compared to WT, the immunoblot analysis of the *Ldhb* KO mice revealed increased expression of tumor necrosis factor (TNF)-α, nuclear factor Nf-ĸB, and inducible NO synthase (NOS2) in the cortex and hippocampal brain homogenates (Figure 6C). Fluorescence microscopy of the brain slices also revealed an increase in IL-1β immunoreactivity in the LDHB deficient mice (Figure 6D). Nonetheless, osmotin treatment significantly reduced the expression of inflammatory mediators (TNF-α, Nf-ĸB, and NOS2) (Figure 6C) and IL-1β immunoreactivity in the cortical and hippocampal brain regions (Figure 6D).

### 3.7. Osmotin Curtailed Spatial Learning and Memory Deficits Observed in Ldhb KO Mice

**Figure 7:**
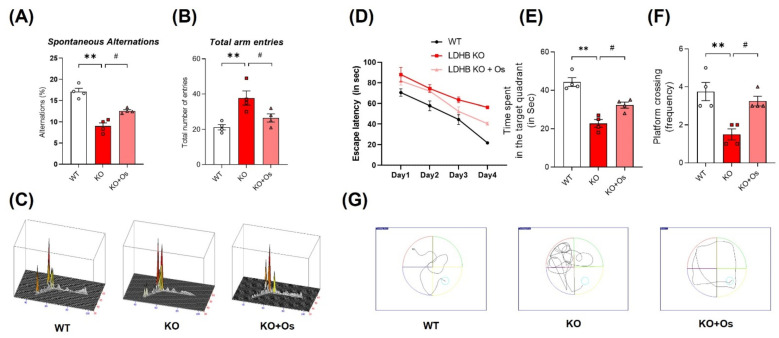
Effect of osmotin on LDHB deficiency-induced cognitive and learning dysfunction. The behavioral studies were performed through the Y-maze test and the Morris water maze (MWM). (**A**,**B**) The graphs represent the % of spontaneous alternation behaviors and the total number of entries in the Y-maze test. The *Ldhb*^−/−^ mice revealed significantly reduced % spontaneous alternation, with a marked difference in the total number of arm entries compared to the WT group. Treatment with osmotin enhanced % spontaneous alternation, signifying improvement in short-term memory and working memory, as well as reduced total number of arm entries in *Ldhb*^−/−^ mice. (**C**) Represents trajectories in the Y-maze test. (**D**) Average escape latency time is taken by mice to reach the hidden platform (from training day 1 to 4 days) in the MWM test. The training period reveals that mice were successful in learning the maze. (**E**,**F**) In the probe test, *Ldhb*^−/−^ mice, compared to WT, illustrated a decline in time spent and an average number of crossing in the arbitrary quadrant in MWM. Osmotin treatment of *Ldhb*^−/−^ increased the total time spent and the number of crossings, implying an improvement in spatial reference and working memory (**G**) Diagram reveals representative trajectories in the MWM test. The data are presented as the mean ± SEM of 4 mice per group, ** *p* ≤ 0.01, ^#^ *p* ≤ 0.05.

Mitochondrial dysfunction is primary and a major contributor to cognitive defects in neurodegenerative disease such as AD. In the brain, mitochondrial impairment causes oxidative stress and bioenergetics deficiency, which aggravates AD disease pathology, leading to synaptic failure and memory loss [42]. Therefore, we determined whether our *Ldhb* KO mice had developed any behavioral deficits. We subjected 24-week-old mice to the Y-maze and MWM test to access spatial working memory and learning/memory functions in all the groups. *Ldhb*^−/−^ mice illustrated insignificant curiosity to explore the novel arms, as revealed by a decrease in % spontaneous alterations, with an increase in total arm entries compared to WT littermates in Y-maze analysis (Figure 7A,B). However, osmotin treated *Ldhb* KO mice displayed higher % spontaneous alterations with the reduced total number of arm entries (Figure 7A,B). Furthermore, to analyze spatial and long-term memory, the mice were exposed to MWM for consecutive four-day acquisition training. All experimental groups featured improvements in learning the maze with time, as indicated by shorter escape latencies in submerged platform training sessions (Figure 7D). However, during a probe test on a 5th day, when the submerged platform was removed, the LDHB deficient mice displayed a decline in time spent and in the number of platform crossing in the target quadrant compared to control littermates (Figure 7E,F). Conversely, osmotin treated *Ldhb* KO mice spent more time and revealed higher numbers of platform crossing compared to the saline-treated LDHB deficient group (Figure 7E,F). As the cognitive decline is directly correlated with synapse loss [43], we then investigated the pre-and post-synaptic protein expression in the cortex and hippocampal brain regions. The *Ldhb* KO mice presented reduced expressions of pre-synaptic synaptosomal-associated protein 23 (SNAP23) and post-synaptic density protein 95 (PSD-95) compared to the WT group (Figure S1A). The immunofluorescence analysis of brain slices also revealed a decrease in PSD-95 immunoreactivity in the cortical and hippocampal-DG region of *Ldhb*^−/−^ mice (Figure S1B). Contrariwise, the osmotin administered *Ldhb* deficient mice revealed upregulated pre-synaptic SNAP23 expression, as well as increased PSD-95 protein expression and immunoreactivity, compared to age-matched control mice (Figure S1A,B). Together, these results illuminate that *Ldhb* KO mice display short-term working memory and spatial learning and retention memory deficits, whilst osmotin treatment alleviates these discrepancies to improve cognitive health in LDHB deficient mice.

## 4. Discussion

In the current study, we generated and used *Ldhb* knockout (*Ldhb*^−/−^) mice. Our findings provide evidence for the role of LDHB deficiency in inducing brain mitochondrial dysfunction associated with oxidative stress, neuroinflammation, and neurodegeneration. We further exhibited that osmotin, an adiponectin natural memetic through AdipoR1, mitigated these discrepancies, possibly, by upregulated expression of p-AMPK/SIRT1/PGC-1α signaling to improve mitochondrial functions and alleviate neuroinflammation and neurodegeneration in *Ldhb*^−/−^ mice.

Although the brain only accounts for 2% of body weight, it consumes 20% of the individual’s total energy. Due to this hefty energy requirement, a slight impairment in the brain bioenergetics may promote various neurodegenerative disorders, such as Alzheimer’s disease (AD) and Parkinson’s diseases (PD) [44]. Neurons are post-mitotic specialized cells, so they consume more than 80% of the total brain energy, and thus, they are inherently reliant on mitochondria and are particularly more vulnerable to a condition such as ageing, in which energy sources are depleted [45,46]. Lactate is an essential source of brain energy metabolism and helps in maintaining neuronal cell survival. The notion is best explained by the astrocyte-neuron lactate shuttle (ANLS) hypothesis [8,9]. LDHB is an important enzyme selectively expressed in neurons [6], and it converts the lactate shuttled from astrocytes to neurons through monocarboxylate transporters (MCTs) to be used as energy metabolite [47], making it important for long-term memory formation [48,49]. We previously reported that loss of LDHB function disrupts the neuronal conversion of lactate into pyruvate, triggers respiratory dysfunction, and suppresses oxidative phosphorylation [12,13], suggesting *Ldhb* gene expression may be directly associated with neuronal energy production and mitochondrial functions. Thus, we interrogated the deficiency of LDHB that might promote brain mitochondrial dysfunction associated with alteration in cellular-redox homeostasis, neuroinflammation, and neurodegeneration in *Ldhb* KO mice.

Mitochondrial impairment and oxidative stress are early symptoms and are commonly implicated in the pathogenesis of several neurodegenerative diseases, including AD [39,42]. Mitochondrial dysfunction causes bioenergetics deficiency and cellular redox dyshomeostasis [50] and, therefore, has detrimental effects on neuronal cells that are critically dependent on mitochondria [51]. Overexpressing *Ldhb* in mice muscle improved mitochondrial gene expression and respiration capacity [52]. On the contrary, suppressing *Ldhb* expression in hepatoma cells disrupted the conversion of lactate to pyruvate, reducing the concentration of ATP production, and causing mitochondrial dysfunction [12]. Similarly, the shRNA-mediated silencing of *Ldhb* in Aβ-resistant PC12 cells displayed an increased mitochondrial ROS production and neurotoxicity [53]. Likewise, the Nrf2-ARE pathway, a modulator of oxidative stress (OS) to keep ROS within the limit, is compromised during aging and neurodegeneration [54]. Nrf2 expression affects cellular bioenergetics, and its deficiency is known to be associated with impaired respiration and reduced ATP levels [55]. Consistent with these, our *Ldhb* deficient mice revealed low ATP levels and a decline in brain endogenous anti-oxidative ability. They also displayed enhanced ROS and LPO production, as well as reduced levels of Nrf2 and cytoprotective enzymes, such as GSH and HO-1, in the cortex and hippocampal regions. These results suggest that LDHB expression is important for mitochondrial bioenergetics, and its deficiency is associated with dysregulated intracellular-redox hemostasis.

Oxidative stress and mitochondrial impairment contribute to neurodegeneration and aging through bioenergetics failure and enhanced ROS accumulation [56]. Being considered as a key regulator of cell death and survival, mitochondria, when losing their structural integrity and function, interact with different proteins to instigate neuronal cell death, leading to neurodegeneration [57]. During apoptosis, mitochondria release various modulators of cell death, including cytochrome-c [58,59], which initiates a series of events, leading to caspase-3 activation, a key executioner of apoptotic cell death [60]. The LDHB deficient mice, which presented dysregulated mitochondrial function, also displayed enhanced neuronal loss, as visualized by increased Bax/Bcl-2 ratio, cytochrome-c release, and upregulated caspase 3 expression. The histological analyses, using cresyl violet staining, further verified the loss of neuronal cells in the cortex and hippocampal brain regions. These results revealed that *Ldhb*^−/−^ mice exhibit a neurodegenerative phenotype.

There is a strong correlation between adiponectin (APN) signaling and mitochondrial bioenergetics [61]. APN mediates AMPK and SIRT1, a metabolic energy sensor through APN-receptors (AdipoR1 and AdipoR2) to facilitate cellular energy homeostasis and regulate mitochondrial functions in different tissues [17,62]. AMPK activation is required to increase both the oxidative capacity and mitochondrial functions in skeletal muscle. Likewise, AMPK-α2 null mice presented decreased expression in PGC-1α [63], a nuclear transcription co-activator and a key player in promoting mitochondrial function and improving oxidative metabolism [24]. Moreover, disrupting AdipoR1 expression in muscle resulted in suppressing AMPK and SIRT1 activation by adiponectin and caused a decrease in PGC-1α expression [17]. Similarly, PGC-1α expression is downregulated in AD brain tissue and other neurodegenerative diseases, indicating impaired mitochondrial impairment [64]. In the current study, *Ldhb* KO mice revealed a decrease in AdipoR1, p-AMPK, and SIRT1 expression. The dysregulated AdipoR1/p-AMPK/SIRT1 axis was associated with reduced PGC-1α activity. Previously, we reported that osmotin and osmotin derived nanopeptide, adipoR1 agonists, regulated brain energy metabolism, and mitigated neuropathological deficits through AdipoR1/AMPK signaling in AD and Adiponectin^−/−^ mice [33,65]). Moreover, we have confirmed that osmotin action is mainly attributed to the activation of AdipoR1, as the silencing of AdipoR1 expression abolished the osmotin ability to activate p-AMPK and SIRT1 in SH-SY5Y neuroblastoma cells [33]. The *Ldhb*^−/−^ mice treated with osmotin (APN homolog) may activate an AdipoR1/p-AMPK/SIRT1 signaling pathway to potentially activate the PGC-1α axis. Hence, we speculate that osmotin probably instigates the p-AMPK/Sirt-1/PGC-1α axis to improve mitochondrial functions in LDHB deficient mice. Correspondingly, p-AMPK/SIRT1/PGC-1α axis activation by osmotin may improve mitochondrial bioenergetics, reduce brain oxidative stress, and reduce cell death and neurodegeneration in *Ldhb* KO mice. PGC-1alpha plays an important role in the suppression of ROS and neuronal loss [28]. Accumulating evidence illustrated the role of PGC-1α in stimulating the expressions of mitochondrial genes and ROS-detoxifying enzymes [66,67]. Activation of AMPK-PGC-1α signaling reduces the hyperglycemia-induced ROS upsurge [67]. Furthermore, endothelial cells, overexpressing PGC1-α, displayed increased mitochondrial membrane potential, reduced ROS, and decreased apoptotic cell death, whilst siRNA mediated downregulation of PGC-1α, which reduced mitochondrial detoxification proteins expression [66]. Moreover, the brain of the PGC-1α null mouse also revealed neurodegenerative lesions [68] and displayed increased sensitivity to ROS-mediated oxidative damage and the neurodegenerative effect of kainic acid and MPTP oxidative-stressors. However, overexpression of PGC-1α protects the cultured neural cells from oxidative-stress-mediated death [28]. These studies collectively demonstrate the role of PGC-1α as a master player in regulating the mitochondrial function and anti-oxidant response against cell death; instigating the AdipoR1/p-AMPK/PGC-1α axis through natural plant protein such as osmotin might play an important role in alleviating mitochondrial alterations and oxidative stress concomitant with neurodegeneration in *Ldhb* deficient mice.

OS has been reported to trigger glial cell immune response, which initiates multiple signaling pathways to induce the release of inflammatory mediators and cytokines such as IL-1β, iNOS, COX2, and TNF-α [69,70]. Activation of astrocytes and microglia, to mediate neuroinflammation, are important components of neurodegenerative diseases [71]. Our LDHB deficient mice revealed astrocytosis and microglial cell activation and showed elevated expression of various inflammatory mediators, including TNF-α, COX2, NOS-2, Il-1β, and Nf-ĸB. Importantly, osmotin attenuated inflammatory response in LDHB deficient mice. Previously, we reported that osmotin attenuates LPS-induced neuroinflammation via regulating the NFκB signaling pathway [72]. Moreover, osmotin has been shown to mimic the anti-inflammatory activity of adiponectin in murine models of colitis [73,74], and it has been reported to be critical in the suppression of vascular inflammation and atherosclerosis [75].

Mitochondrial dysfunction, associated with oxidative damage, causes synapse loss and impairs neurotransmission, promoting cognitive deficits, and is predictive of the aging phenotype [76]. The neuronal ability to produce sufficient ATP is impaired during aging and neurodegenerative disorders, provoking synapse failure and neural degeneration [77]. Freshly isolated synaptosomes from the adult rat brain have revealed an increase in LDHB enzyme expression [78]. Furthermore, accumulative evidence illuminated the role of lactate, produced and transported between astrocytes and neurons through MCTs, which is required for long-term memory formation [48]. Importantly, our study revealed that *Ldhb* KO mice have a decline in memory performance. LDHB deficient mice displayed spatial working and retention memory deficits, as visualized in both Y-maze and Morris Water Maze (MWM) test, compared to the age-matched wild type. Moreover, the behavioral impairments in *Ldhb* KO mice were also correlated with a decline in cortical and hippocampal pre-synaptic synaptosomal-associated protein 23 (SNAP23) and postsynaptic density protein 95 (PSD-95) expression when compared to the WT group. APN has been reported to alleviate neurobehavioral deficits in aged mice after cerebral ischemia [79]. Similarly, chronic APN deficiency in aged mice leads to cognitive impairments, through attenuated APMK signaling [80], and exacerbates neuronal loss and cognitive deficits in 5xFAD mouse [81]. Previously, we reported that suppression of AdipoR1 promotes metabolic dysfunction associated with AD-like pathology and memory deficit [82], while intraperitoneal injection of osmotin alleviated neuropathological and neurobehavioral deficits in APP/PS1 mice [33]. Similarly, the expression of PGC-1α is reduced, in AD, as a function of clinical dementia [83]. In line with these, our LDHB deficient mice, treated with osmotin, reduced neurobehavioral impairments and improved synaptic functions.

## 5. Conclusions

**Figure 8:**
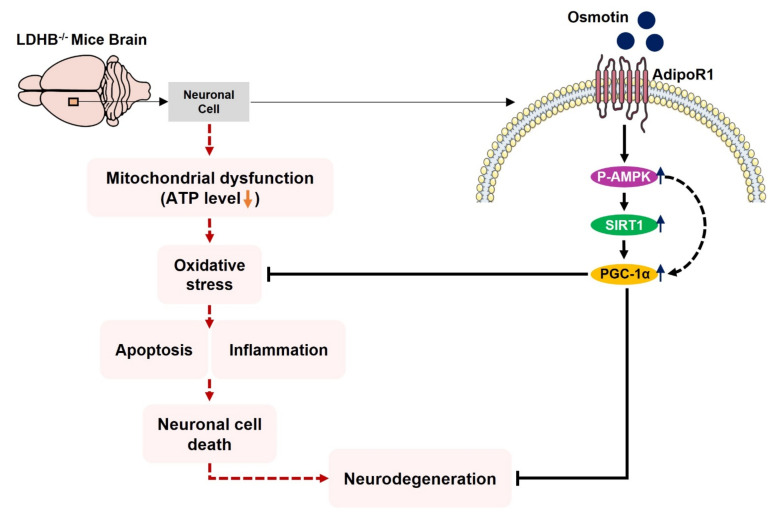
Schematic illustration of proposed detrimental consequences of LDHB deficiency on brain mitochondrial function and neurodegeneration. The schematic diagram shows that the deficiency of LDHB in the brain can induce neurodegeneration through mitochondrial dysfunction. Using osmotin improved mitochondrial bioenergetics and suppressed ROS induced oxidative stress, neuroinflammation, and neurodegeneration, probably by upregulating the p-AMPK/Sirt1/PGC-1α signaling pathway.

We conclude, to the best of our knowledge, that *Ldhb*^−/−^ mice presented brain mitochondrial impairment, oxidative stress associated with neuroinflammation and revealed neurodegenerative phenotype and synapse loss/cognitive deficit. However, treatment with osmotin alleviated brain oxidative stress, neuroinflammation, and reduced neuronal degeneration, as well as improved neurobehavioral measures. Importantly, our model may present significant implications for pathologies associated with mitochondrial dysfunction/oxidative stress, including Alzheimer’s disease, aging, and other neurodegenerative conditions. A proposed simple schema to illuminate the adverse consequences of LDHB deficiency associated with brain oxidative stress and neurodegeneration, along with the potential therapeutic effect of osmotin, is depicted in Figure 8.

## Figures and Tables

**Table 1:** List of primary and secondary antibodies.

Primary Antibodies	Dilution	Source	Identifier
Anti-LDHB	IB = 1:1000, IF = 1:200, IHC = 1:200	Invitrogen	PA5-27505
Anti-Nrf2	IB = 1:1000, IF = 1:500	Santa Cruz	SC-365949
Anti-HO-1	IB = 1:1000, IF = 1:200	Santa Cruz	SC-136960
Anti-Bax	IB = 1:1000	Santa Cruz	SC-7480
Anti-Bcl-2	IB = 1:1000	Santa Cruz	SC-7382
Anti-caspase-3	IB = 1:1000, IF = 1:200	Santa Cruz	SC-56052
Anti-Cytochrome C	IB = 1:1000	Santa Cruz	SC-8385
Anti-AdipoR1	IB = 1:1000	Abcam	Ab-126611
Anti-P-AMPK	IB = 1:1000	Abcam	#2535
Anti-AMPK	IB = 1:1000	Abcam	#2532
Anti-Sirt1	IB = 1:1000	Santa Cruz	SC-74465
Anti-PGC-1α	IB = 1:1000, IF = 1:200	Santa Cruz	SC-13067
Anti-Tnf-α	IB = 1:1000	Santa Cruz	SC-52746
Anti-p-NF-ĸB	IB = 1:1000	Santa Cruz	SC-136548
Anti-NOS-2	IB = 1:1000, IF = 1:200	Santa Cruz	SC-52746
Anti-IL-1β	IF = 1:200	Santa Cruz	sc-32294
Anti-GFAP	IB = 1:1000, IF = 1:200	Sigma	G-3893
Anti-Iba-1	IB = 1:1000	Wako chemicals	16-20001
Anti-SNAP-23	IB = 1:1000	Santa Cruz	SC-166244
Anti-PSD-95	IB = 1:4000, IF = 1:500	Cell signaling	3450
Anti-β-actin	IB = 1:1000	Santa Cruz	SC-47778
Anti-Mouse IgG	IB = 1:2500	Promega	W4021
Anti-Rabbit IgG	IB = 1:2500	Promega	W4011
Biotinylated mouse IgG	IHC = 1:100	Santa Cruz	SC-2762
Goat anti-mouse IgG-FITC	IF = 1:50	Invitrogen	A32723
Goat anti-rabbit IgG-FITC	IF = 1:50	Invitrogen	A32732

## Data Availability

The data is contained within the article and supplementary material.

## Supplementary Materials

The following are available online at https://www.mdpi.com/article/10.3390/antiox11020261/s1, Figure S1: Osmotin enhanced synaptic protein expression.

## Abbreviations

APN	Adiponectin
AdipoR1	Adiponectin Receptor 1
AD	Alzheimer’s disease
AMPK	AMP-activated protein kinase
ANOVA	Analysis of variance
ATP	Adenosine triphosphate
DAPI	4′, 6′-Diamidino-2-phenylindole
FITC	Fluorescein isothiocyanate
GSH	Reduced glutathione
GSSG	Glutathione disulfide
IP	Intraperitoneal
LDHB	Lactate dehydrogenase B
LPO	lipid peroxidation
MPTP	1-methyl-4-phenyl-1,2,3,6-tetrahydropyridine
MWM	Morris water maze
PBS	Phosphate-buffered saline
PGC-1α	Peroxisome proliferator-activated receptor gamma coactivator 1-alpha
ROS	Reactive oxygen species
SIRT1	NAD-Dependent Protein Deacetylase Sirtuin-1
TRITC	Tetramethyl rhodamine isothiocyanate
WT	Wild type
