# Supplementary material for: LDHB Deficiency Promotes Mitochondrial Dysfunction Mediated Oxidative Stress and Neurodegeneration in Adult Mouse Brain
Source: Antioxidants (Basel). 2022 Jan 28;11(2):261. doi: 10.3390/antiox11020261 (PMC8868245; doi:10.3390/antiox11020261)
Supplement: Supplementary file 1 [file antioxidants-11-00261-s001.zip › antioxidants-1552442-supplementary.pdf]

Supplementary Information for

LDHB deficiency promotes mitochondrial dysfunction mediated oxidative stress and neurodegeneration in adult mouse brain

Jun Sung Park[1†], Kamran Saeed[1†], Myeung Hoon Jo[1], Min Woo Kim[1], Hyeon Jin Lee[1], Chan-Bae Park[2], Gwang Lee[3], Myeong Ok Kim[1,4,*]

[1] Division of Life Science and Applied Life Science (BK21 FOUR), College of Natural Sciences,

Gyeongsang National University, Jinju, 52828, Republic of Korea

[2] Department of Otolaryngology, Ajou University School of Medicine, Suwon,16499, Korea; pcbkaist@gmail.com or chanbaepark@ajou.ac.kr or chanbaepark@ajou.ac.kr (C.-B.P.)

[3] Department of Physiology, Ajou University School of Medicine, Suwon 16499, Republic of Korea; glee@ajou.ac.kr (G.L.)

[4] Alz-Dementia Korea Co., Jinju 52828, Korea

Correspondence and requests for materials should be addressed to M.O.K (e-mail: mokim@gnu.ac.kr)

Supplementary Figure and Legend

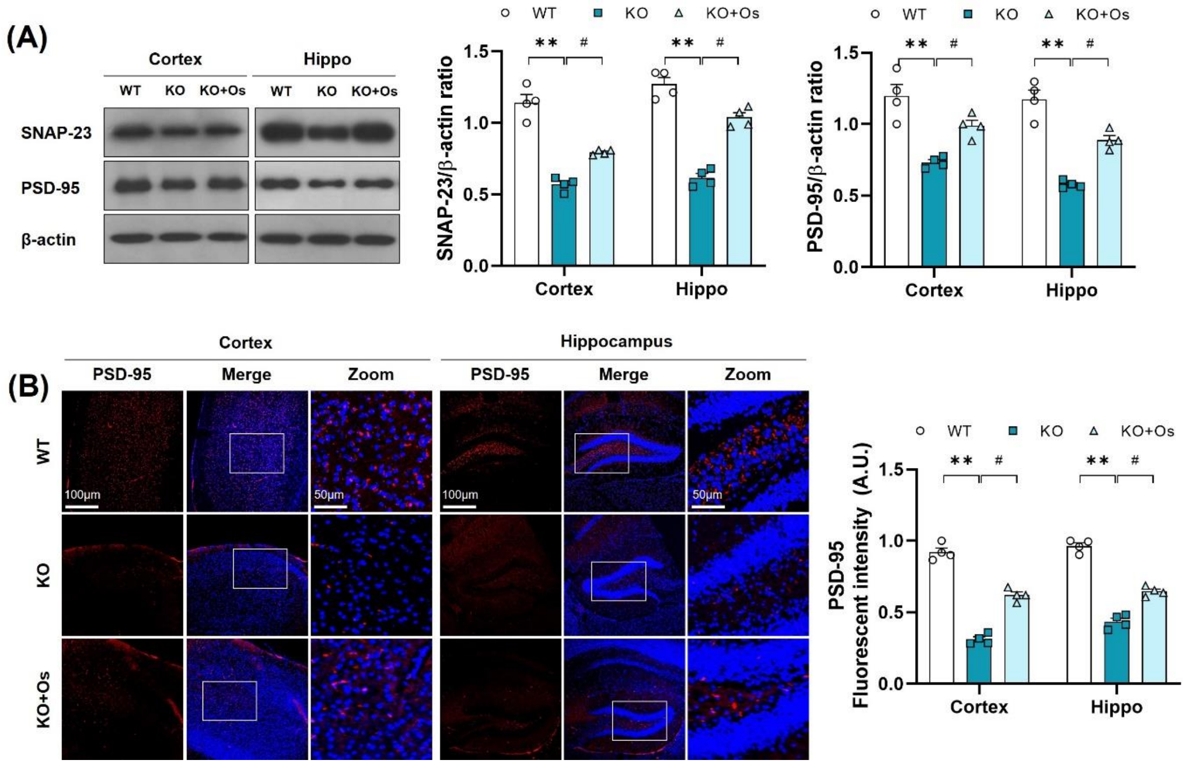

Figure. S1. Osmotin enhanced synaptic protein expression. **(A)** Western blot analysis of SANP-23 and PSD-95 in the cortex and hippocampus of adult mouse brains. The bands were quantified using ImageJ software, and the differences are represented by histograms. β-actin was used as a loading control. **(B)** Confocal immunofluorescence photomicrographs of PSD-95 reactivity in the cortex and hippocampus of the experimental groups. The density values are relative to the WT group and expressed in arbitrary units (A.U.). The data are presented as the mean ± SEM of 4 mice per group, ** $p \leq 0.01$, $^{\#}$ $p \leq 0.05$.
